# Osteopontin from monocyte-derived dendritic cells mediates ozone-induced pulmonary responses in mice

**DOI:** 10.3389/fimmu.2026.1748667

**Published:** 2026-03-19

**Authors:** Hiroki Tashiro, Yuki Kuwahara, Yuki Kurihara, Yoshie Konomi, Ayako Takamori, Shinya Kimura, Koichiro Takahashi

**Affiliations:** 1 Division of Hematology, Respiratory Medicine and Oncology, Department of Internal Medicine, Faculty of Medicine, Saga University Hospital, Saga, Japan; 2 Clinical Research Center in Hiroshima, Hiroshima University Hospital, Hiroshima, Japan

**Keywords:** airway hyperresponsiveness, dendritic cells, neutrophils, osteopontin, ozone

## Abstract

**Introduction:**

Asthma is a heterogeneous disease characterized by airway inflammation and hyperresponsiveness (AHR). Ozone, a common air pollutant, induces a neutrophilic phenotype of asthma that is resistant to corticosteroids. Osteopontin has been implicated in airway inflammation, but its role in ozone-induced pulmonary responses remains unclear. We hypothesized that osteopontin contributes to neutrophilic airway inflammation and AHR through dendritic cell (DC)–neutrophil interactions.

**Methods:**

Male BALB/c mice were exposed to ozone (2 ppm, 3 hours) or ambient air. AHR and bronchoalveolar lavage fluid (BALF) cell counts were assessed, and osteopontin levels were measured. Single-cell RNA sequencing of lung tissue was performed to identify osteopontin-producing cells and infer cell–cell communication using CellChat analysis. Functional validation included administration of clodronate liposomes to affect monocyte-derived DCs and an anti-osteopontin neutralizing antibody. In addition, RNA sequencing of bone marrow-derived neutrophils stimulated with osteopontin *in vitro* was conducted.

**Results:**

Ozone exposure significantly increased neutrophils in BALF, enhanced AHR, and elevated osteopontin levels in lung tissue. Single-cell RNA sequencing showed DCs, particularly monocyte-derived DCs, as the main source of osteopontin, with downstream signaling to macrophages, fibroblasts, pericytes, smooth muscle cells, and neutrophils. Clodronate liposome treatment reduced osteopontin expression and attenuated ozone-induced neutrophilic inflammation and AHR. Neutralization of osteopontin suppressed neutrophilic airway inflammation but did not significantly improve AHR. *In vitro*, osteopontin stimulation of neutrophils upregulated *NDUFA1*, a mitochondrial complex I gene.

**Conclusions:**

The present findings indicate that ozone exposure induces osteopontin production primarily from monocyte-derived DCs, which promotes neutrophilic airway inflammation through DC–neutrophil interactions, while having a limited impact on airway hyperresponsiveness.

## 1 Introduction

Asthma is clinically characterized by airway inflammation and airway hyperresponsiveness (AHR), which causes variable limitation of airflow by paroxysmal airway narrowing (1, 2). The clinical features are considered to be induced by external triggers such as allergens, pollens, viral infections, cigarettes, and pollutants, and they are recognized by dendritic cells (DCs) and interact with specific lymphocytes such as helper T cells (3, 4). As the alternative pathway, epithelial cell-derived cytokines such as thymic stromal lymphopoietin (TSLP) and interleukin 33 (IL-33), which are induced by cell damage caused by the triggers, activate DCs and innate lymphoid cells type 2 (ILC2s) to induce the pathophysiology of asthma (5). The pivotal treatment of asthma is inhaled corticosteroids, but a few patients with a refractory phenotype need systemic corticosteroid therapy, which reduces the mortality of patients with a poor clinical course of asthma, but the side effects of corticosteroids accumulate (6, 7). Recently, several biologics targeting inflammatory pathways such as immunoglobulin E, IL-4, IL-5, IL-13, and TSLP have become available and have the capacity to improve the pathophysiology of severe eosinophilic asthma (type 2-dominant phenotype that includes worsened pulmonary function, symptoms, and exacerbations) with fewer adverse effects caused by the decreased corticosteroid dose (8, 9). However, the efficacy of these drugs depends on the individual, because the inflammatory phenotypes of the airways are heterogeneous, and patients with the low or non-type 2 inflammatory phenotype cannot generally be controlled. Therefore, identification of other molecules reflecting the pathophysiology of asthma, such as AHR and airway inflammation, especially when eosinophilic inflammation is lacking, is increasingly demanded to explore future therapeutic targets for patients with severe asthma with non-type 2 inflammation.

Ozone, an air pollutant, is one of the triggers of asthma, and several observational studies have demonstrated that an increased concentration of ozone in the atmosphere is associated with exacerbation of asthma pathophysiology, such as decreased pulmonary function and elevated asthma attack rates (10, 11). In mice, ozone at 2 ppm for 3 hours increases neutrophilic airway inflammation and AHR, a certain inflammatory phenotype of asthma called low or non-type 2 inflammation (12, 13). Lung immune cells reflecting pulmonary responses to ozone that are considered crucial include macrophages, γδ T cells, ILC2s, and DCs, and these cells regulate inflammatory cytokines such as tumor necrosis factor alpha (TNFα), IL-17A, IL-33, and TSLP (14–16). Furthermore, recent developments of experimental techniques such as single-cell RNA sequencing and comprehensive bioinformatic analyses including cell–cell communication allow us to explore targeted cell clusters and genes reflecting pathophysiology in disease models (17, 18). According to these findings, exploring targeted molecules that reflect ozone-induced pulmonary responses, characterized by non-eosinophilic airway inflammation and AHR, might be feasible using this comprehensive experimental method to identify new therapeutic targets for patients with severe asthma with non-type 2 inflammation.

Osteopontin is one of the inflammatory cytokines produced by various immune cells, and it is involved in various clinical pathophysiological processes related to bone remodeling, tumorigenesis, inflammatory responses, and cell proliferation and migration in patients with rheumatoid arthritis, cancers, cardiovascular diseases, and asthma (19–23). In patients with asthma, osteopontin levels in serum and bronchoalveolar lavage fluid (BALF) were higher than in healthy individuals, and they were strongly associated with disease severity (24). In addition, increased expression of osteopontin in airways was also associated with decreased pulmonary function, which is one of the characteristics of severe asthma (25). In mice, exposure to viral infection increased osteopontin expression in the airways, contributing to augmented airway inflammation and AHR (26). These data showed that osteopontin might contribute to airway inflammation and AHR in asthmatic mouse models, but the association with ozone-induced pulmonary responses and the mechanistic clarification, including the producing cells, are not fully understood. We hypothesized that osteopontin is involved in pulmonary responses to ozone, including neutrophilic airway inflammation and AHR. Then, single-cell RNA sequencing analysis was performed to identify the source of osteopontin and the interacting signals in lung cells. Finally, exploratory analyses of experiments using neutrophils stimulated with osteopontin were done to demonstrate the possible mechanisms of osteopontin in neutrophilic airway inflammation.

## 2 Materials and methods

### 2.1 Mice and experimental protocols

BALB/c mice were purchased from Japan SLC (Shizuoka, Japan). For all experiments, male 7- to 9-week-old mice housed at the Saga University Animal Facility under specific pathogen-free conditions with a 12-hour light/12-hour dark cycle were used. Mice were maintained with free access to drinking water and food. Animal experiments were conducted in accordance with the guidelines for the care and use of experimental animals by the Japanese Association for Laboratory Animals Science (1987), and all protocols were approved by the Saga University Animal Care and Use Committee. To evaluate pulmonary responses to ozone, male BALB/c mice were exposed to ozone at 2 ppm or ambient air for 3 hours. Examination of AHR and collection of BALF were performed 24 or 48 hours after air or ozone exposure, referring to previous reports (27, 28). To focus on the functions of monocyte DCs and macrophages in airway responses to ozone, 100 μL of clodronate liposome (100 mg/mL, #16001004; HYG, The Netherlands) or control liposome (100 mg/mL, #16003632; HYG) were administered intranasally to BALB/c mice 72 hours before ozone or air exposure. For neutralization of osteopontin, 100 μL of anti-mouse osteopontin antibody (2 g/mL, #BE0372; Bio X Cell, USA) as osteopontin inhibitor or mouse IgG2c isotype control (2 g/mL, #BE0366; Bio X Cell) as placebo were administered subcutaneously for 4 consecutive days, and ozone or ambient air exposure was performed 2 hours after the final injection.

### 2.2 Ozone and ambient air exposure

Mice were exposed to ozone at 2 ppm for 3 hours in a sealed chamber with detailed monitoring of the concentration of ozone, which was mixed with pressurized room air, referring to the previous reports (12, 16). Briefly, the concentration was strictly regulated by an ozone monitor between 1.8 and 2.2 ppm, and control mice were placed in the same type of sealed chamber with exposure to ambient air. During exposure to ozone or ambient air, mice could not access food and water, but they were immediately returned to their cages with free access to food and water after exposure.

### 2.3 Measurement of pulmonary mechanics, airway responsiveness, and collection of bronchoalveolar lavage fluid

Mice were anesthetized using pentobarbital and xylazine, and an 18-gauge metal needle was inserted into the trachea to measure AHR with the forced oscillation technique by connecting to a flexiVent system (SCIIREQ, Montreal, Canada). First, a positive end-expiratory pressure of 3 cmH_2_O was applied. To ensure a consistent volume history, three inflations to total lung capacity (corresponding to a trans-respiratory system pressure of 30 cmH_2_O) were performed. One minute after the final inflation to total lung capacity, 10 breaths of aerosolized phosphate-buffered saline (PBS) were administered. Subsequently, total respiratory system resistance (Rrs) was measured using the forced oscillation technique at 15-second intervals for a duration of 3 minutes. The same sequence was then repeated with aerosolized methacholine chloride at progressively increasing concentrations of 1, 3, 10, 20, and 50 mg/mL. For each concentration, the three highest Rrs values were averaged to generate the dose–response curves to methacholine, referring to previous reports (13, 29). After the measurement of AHR, BALF samples were obtained as previously described (30, 31). Briefly, BALF was obtained by lavage with 1 mL of PBS, and the procedure was performed twice. The total cell number was determined by a hemocytometer, and cell differentiation was assessed by counting at least 300 cells in samples stained with Diff-Quik.

### 2.4 Lung histology

Histological analyses were performed as previously described (30). Lung tissues were fixed in 10% neutral buffered formalin, paraffin-embedded, and sectioned at a thickness of 4 μm. The sections were stained with hematoxylin and eosin (H&E). Digital images of the stained sections were acquired using a NanoZoomer S60 scanner and examined with NDP.view.2 software (Hamamatsu Photonics K.K., Hamamatsu, Japan).

### 2.5 Preparation of lung homogenate and measurement of cytokine and chemokine levels

The right lung was homogenized by TissueLyser LT (QIAGEN, Hilden, Germany) oscillating at 50 Hz for 5 minutes with digestion buffer containing 50 mM Tris-buffered saline (pH 8.0) containing 1.0% Triton X-100, 0.1% sodium dodecyl sulfate, 150 mM NaCl, 0.5% sodium deoxycholate, 1 mM phenylmethylsulfonyl fluoride, 1 μg/mL aprotinin, 1 μg/mL leupeptin, and 1 mM Na_3_VO_4_. The samples were then centrifuged at 10,000g for 15 min, and supernatants were obtained and stored at -80 °C until the analyses. Concentrations of osteopontin in lung tissue samples were measured by an enzyme-linked immunosorbent assay kit (R&D Systems, Minneapolis, MN, USA) according to the manufacturer’s instructions. A commercial based multiplex assay (Mouse Cytokine/Chemokine 31-Plex Discovery Assay Array; Eve Technologies, Calgary, Canada) was used to measure other cytokines and chemokines in lung tissue samples. The limit of detection for the cytokines and chemokines in the multiplex assay was 0.64 pg/mL.

### 2.6 Purification of neutrophils from bone marrow cells and stimulation with osteopontin

Purification of neutrophils from bone marrow cells was as described in a previous report (32). Briefly, bone marrow cells were collected from femora and tibiae of BALB/c mice by flushing with RPMI 1640 medium (Thermo Fisher Scientific) supplemented with 10% fetal bovine serum, 100 IU/mL penicillin, 0.1 mg/mL streptomycin, and 2 mM ethylenediaminetetraacetic acid. Histopaque-1119 and Histopaque-1077 (Sigma-Aldrich, St. Louis, MO, USA) were gently placed on the obtained bone marrow cells. Purified neutrophils were obtained from the stratified layer between the Histopaque-1119 and Histopaque-1077 by the concentration gradient method. Diff-Quik staining of the cells in the stratified layer, followed by microscopic visualization indicated that approximately 90% of these cells were neutrophils. In addition, 1.0x10^6^ bone marrow neutrophils were stimulated with 1.0 μg/mL of mouse recombinant osteopontin (R&D Systems) in a 24-well dish for 2 hours, referring to a previous report (33), and collected for further RNA extraction and flowcytometric analyses.

### 2.7 Extraction of RNA and RNA sequencing analysis of bone marrow neutrophils

RNA was extracted from bone marrow neutrophils using the RNeasy mini-Kit (QIAGEN, Hilden, Germany). Quantity and quality were checked using a DS-11 Spectrophotometer (DeNovix, Wilmington, DE, USA). For RNA sequencing analysis, total RNA was extracted from 12 samples of 6 bone marrow neutrophils without stimulation and 6 bone marrow neutrophils with osteopontin stimulation. Only RNA samples passing quality control were used for downstream library preparation. RNA-seq libraries were prepared using the TruSeq Stranded mRNA Library Prep Kit (Illumina, San Diego, CA, USA), which enriches for polyadenylated transcripts and maintains strand specificity. All procedures were carried out according to the manufacturer’s protocol at Rhelixa Inc. (Tokyo, Japan). Prepared libraries were sequenced on an Illumina NovaSeq X Plus platform, generating paired-end reads with a length of 150 base pairs. For each sample, approximately 20 million pairs were obtained, corresponding to 6 gigabases of sequencing data. Sequenced reads were demultiplexed and aligned with the database of the mouse reference genome (mm39: GRCm39). Statistical analysis and visualization were performed by R [4. 4. 2] with tidyverse [2. 0. 0], Rsubread [2. 20. 0], DESeq2 [1. 46. 0], pheatmap [1. 0. 13], ggrepel [0. 9. 6], EnhancedVolcano [1. 24. 0], org.Mm.eg.db [3. 20. 0], AnnotationDbi [1. 68. 0], clusterProfiler [4. 14. 6], msigdbr [25. 1. 1], and R.utils [2. 13. 0].

### 2.8 Flow cytometric analysis of bone marrow neutrophils and lung single-cell suspension

Bone marrow neutrophils with or without stimulation by osteopontin were stained with CD11a (#101119, M17/4, BioLegend, San Diego, CA, USA), CD11b (#15011282, M1/70, Thermo Fisher Scientific, Waltham, MA, USA), and CD49d (#103635, R1-2, BioLegend) for the functional analysis of cell adhesion. In terms of lung single-cell analysis, following the removal of red blood cells by perfusion with PBS, lung tissue was excised, minced into small fragments, and passed through a 70-μm strainer into a digestion buffer containing 0.02 mg/mL deoxyribonuclease I (Thermo Fisher Scientific) and 0.7 mg/mL collagenase type II (Worthington Biochemical Corporation, Lakewood, NJ, USA). Residual erythrocytes were eliminated using BD Pharm Lyse buffer (BD Bioscience, Franklin Lakes, NJ, USA), yielding a single-cell suspension. The suspensions were then pretreated with an FcγR-blocking antibody (BD Bioscience) and washed prior to staining. For flow cytometric analysis (FACS Verse; BD Bioscience), cells were stained with the following antibodies: CD45 (#20-0451-U100, 30-F11, TONBO Biosciences, Tucson, AZ, USA), MHC-II (#107620, M5/114.15.2, BioLegend), CD64 (#139303, X54-5/7.1, BioLegend), and CD11b (Thermo Fisher Scientific). Data were subsequently analyzed using FlowJo software (Tree Star, Ashland, OR, USA).

### 2.9 Single-cell RNA sequencing of lung tissue

The detailed method of single-cell RNA sequencing was previously reported (16). Briefly, lung tissues of air-exposed mice (n = 4) and ozone-exposed mice (n = 4) were dissected and transferred through a mesh with digestion medium consisting of collagenase type 2, deoxyribonuclease, dispase, and elastase. Then, a single-cell suspension was obtained. On flowcytometry analysis, cells were stained with 7AAD, CD45, and CD45-positive cells, and CD45-negative cells were sorted by a cell sorter (MA900, Sony, Tokyo, Japan) after elimination of dead 7AAD-positive cells. After confirming viability by trypan blue staining (> 85%), the cells were loaded on a Chromium Next GEM Single Cell 3′ Kit v3.1 controller (10x Genomics, Pleasanton, CA, USA) to generate single-cell gel beads in emulsion according to the manufacturer’s instructions at Rhelixa Inc. (Tokyo, Japan). Sequenced reads generated from the NovaSeq 6000 were demultiplexed and aligned with the database of the mouse reference genome (mm10: GRCm38). The present samples of single-cell RNA sequencing are included in the previous reported dataset (16). However, sequence data of air-exposed mice and ozone-exposed mice were newly aggregated, and downstream single-cell analyses were performed once again by Cell Ranger (version 8. 0. 1, 10x Genomics, https://www.10xgenomics.com/jp/products/cloud-analysis) containing transcriptome lengths of 150 base pairs, 2 paired-end, 120 gigabases, and 800 million reads per samples. Additional bioinformatic analyses including annotation, gene expression comparison, and cell–cell communication were also newly performed.

### 2.10 Bioinformatic analysis for annotation, gene expression comparison, and cell–cell communication in each cell cluster on single-cell RNA sequencing

For annotation of cell clusters, major gene markers were considered to perform identification of specific cells along with general reorganization of component cells depending on whether the sorted cells were CD45-positive or CD45-negative. Briefly, in terms of CD45-negative cells, gene expression levels of *Ptprc*, *Pecam1*, *Acta2*, *Pdgfra*, *Col13a1*, *Sftpc*, *Pdpn*, *Scgb1a1*, and *Pdgfrb* and the top 50 upregulated gene expressions depending on the adjusted p-value of the targeted cell cluster compared with the entire dataset were considered for annotation. In terms of the CD45-positive cells, gene expression levels of *Ptprc*, *Cd3e*, *Cd19*, *Ncr1*, *Itgax*, *Itgam*, *Foxp3*, and *Il1rl1* and the top 50 upregulated gene expressions depending on the adjusted p-value of the targeted cell cluster compared with the entire dataset were considered for annotation. For sub-clustering of dendritic cells, annotation of dendritic cell clusters was performed under a re-clustering system, and further annotation was determined by gene expression levels of *Itgax*, *Itgam*, *Batf3*, *Irf2*, *H2-Ab1*, *Tcf4*, and *Klf4* and the top 50 upregulated gene expressions depending on the p-value of the targeted cell cluster compared with the entire dataset in dendritic cell clusters. All of the visualizations and comparisons of gene expressions were performed by Loupe Browser 8 (10x Genomics, https://www.10xgenomics.com/jp/support/software/loupe-browser). In terms of cell–cell communication, the developed tool called CellChat, which can analyze intercellular communication networks from single-cell RNA sequencing data, was used, referring to a previous report (17). Briefly, this tool generates a comprehensive database of signaling molecule interactions that are systematically curated by incorporating the known structural characteristics of ligand–receptor interactions. This includes multimeric ligand–receptor complexes, soluble agonists and antagonists, and stimulatory and inhibitory membrane-bound co-receptors. Based on this framework, CellChat infers cell state–specific signaling communication from single-cell RNA sequencing data by applying mass action models, together with differential expression analysis and statistical testing of cell groups. The signal strength between cell groups was computed based on the expression of ligand–receptor pairs. Specifically, the average expression levels of ligands in the sender population and receptors in the receiver population were combined to derive interaction scores, with appropriate adjustments for multi-subunit complexes. The resulting scores were normalized by cell population size and aggregated across ligand–receptor pairs to quantify the overall signaling strength of each pathway. The network diagram shows the predicted cell–cell communication between different cell types identified by single-cell RNA sequencing. Each node represents a specific cell type, and the lines represent predicted ligand–receptor interactions. The thickness of the lines indicates the strength of interactions, whereas colors distinguish the different cell types. Statistical analysis and visualization were performed by R [4. 4. 2], with Seurat [5. 3. 0], CellChat [2. 1. 2], ggplot2 [3. 5. 2], dplyr [1, 1, 4], and future [1. 67. 0].

### 2.11 Data availability

The raw data for single-cell RNA sequencing and normal RNA sequencing have been deposited in the DNA Data Bank of Japan (https://www.ddbj.nig.ac.jp/index.html) under accession numbers DRA017579 and DRA022501, respectively.

### 2.12 Statistical analysis

Differences between two groups were analyzed by Student’s *t*-test or the Mann-Whitney U test. One-way analysis of variance was used for multiple comparisons of continuous variables. When a significant difference was identified, the *post hoc* Dunnett test was used for each group comparison. On single-cell RNA sequencing analysis, the significance of gene expression levels in each cell cluster was evaluated by the Benjamini-Hochberg correction for multiple comparisons, and adjusted p-values were generated for the top 5 significant genes in each cell cluster. On RNA sequencing of bone marrow neutrophils, exploratory pathway enrichment analysis was performed using genes with a nominal p < 0.05 considered significant, because differential expression analysis identified only a small number of genes with an adjusted p-value less than 0.05. Other comparisons in differential expression analysis based on the negative binomial distribution were evaluated by the Benjamini-Hochberg correction for multiple comparisons, and adjusted p-values were generated to assess significance. Principal component analysis (PCA) was performed on variance-stabilized expression data using a multivariate statistical method based on Euclidean distance. Other detailed methods of statistical analysis in balk RNA sequencing and single cell RNA sequencing were mentioned previously. Significance was set at p < 0.05. Data were analyzed using JMP Pro version 14 (SAS Institute Japan, Tokyo, Japan) and GraphPad Prism version 10 (GraphPad Software, Boston, MA, USA).

## 3 Results

### 3.1 Ozone exposure induces neutrophilic airway inflammation and airway hyperresponsiveness with increased osteopontin concentrations in the lungs

**Figure 1:**
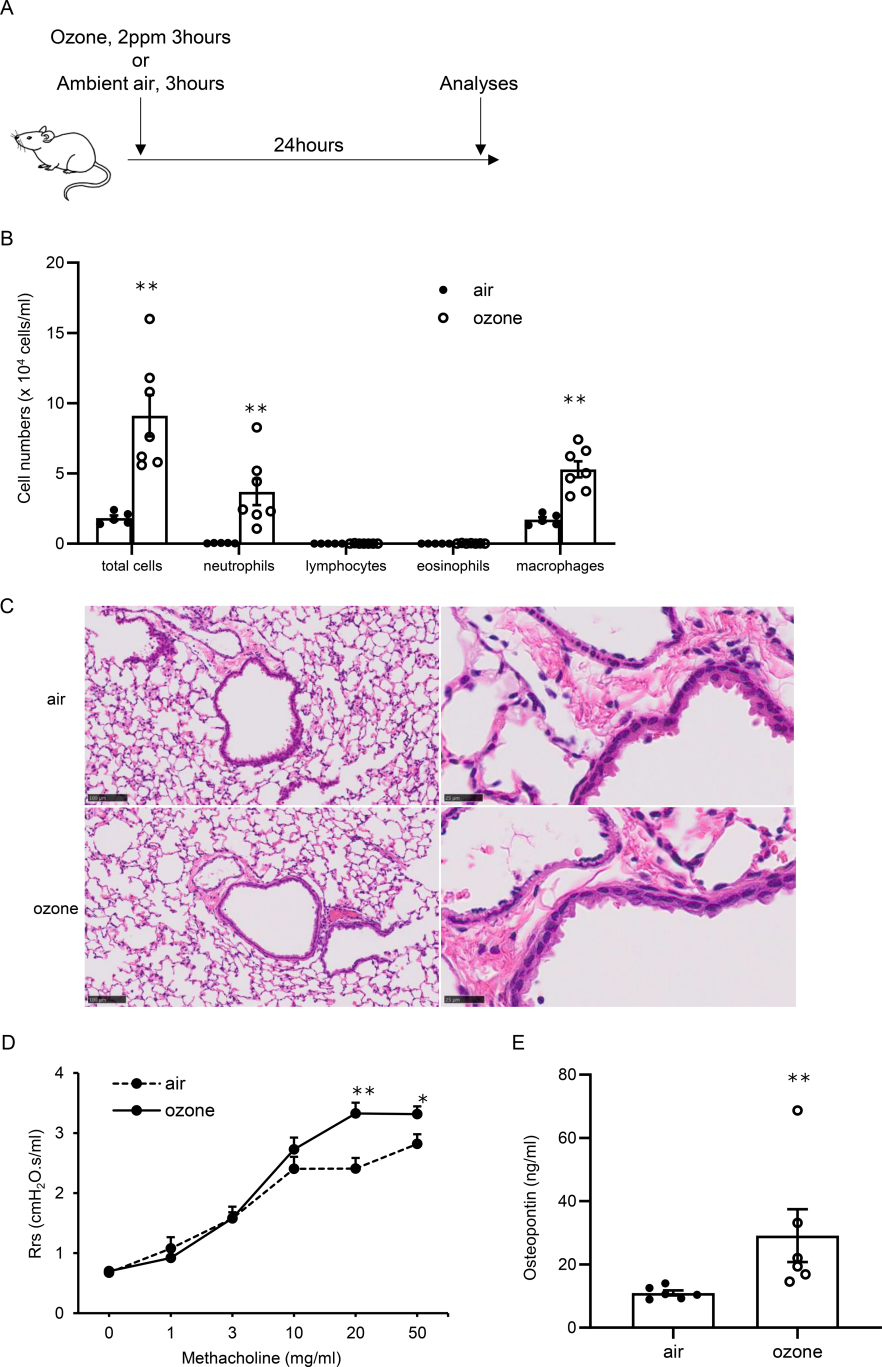
Cell counts in bronchoalveolar lavage fluid (BALF), airway hyperresponsiveness, and concentrations of osteopontin in lungs of mice exposed to air or ozone. **(A)** Experimental protocol. **(B)** Results of cell counts in BALF in mice exposed to air or ozone. **(C)** Lung histological examination by hematoxylin and eosin staining. **(D)** Results of airway hyperresponsiveness in mice exposed to air or ozone. **(E)** Concentrations of osteopontin in mice exposed to air or ozone. The data shown are pooled from multiple experiments (n = 5–7 per group). Results are means ± standard error. **p < 0.01, *p < 0.05 compared with mice exposed to air.

To evaluate the impact of osteopontin levels in lungs with ozone exposure and the pulmonary response to ozone, mice were exposed to air or ozone, and BALF cell counts, AHR, and osteopontin concentrations in the lungs were examined (**Figure 1A**; **Supplementary Figure S1A**). At 24 hours after ozone exposure, total cell counts, including neutrophils and macrophages, in BALF were significantly higher in mice exposed to ozone than in those exposed to air (**Figure 1B**). Histopathological analysis of the lungs showed no evidence of inflammatory cell infiltration or epithelial damage in the airways of either air-exposed or ozone-exposed mice at this time point (**Figure 1C**). In addition, AHR was greater in mice exposed to ozone than in mice exposed to air (**Figure 1D**). The osteopontin level in the lungs was also higher in mice exposed to ozone than in mice exposed to air (**Figure 1E**). Collectively, these data show that ozone exposure induces neutrophil-dominant airway inflammation, enhances AHR, and increases osteopontin levels in the lungs 24 hours after exposure. At 48 hours after ozone exposure, total cell counts, including neutrophils and macrophages, in BALF were slightly higher in mice exposed to ozone than in those exposed to air (**Supplementary Figure 1B**). However, AHR was not altered in mice exposed to ozone compared with mice exposed to air (**Supplementary Figure 1C**). Based on these findings, all other experiments were performed 24 hours after air or ozone exposure.

### 3.2 Cytokine and chemokine levels in the lungs are altered by ozone exposure

**Figure 2:**
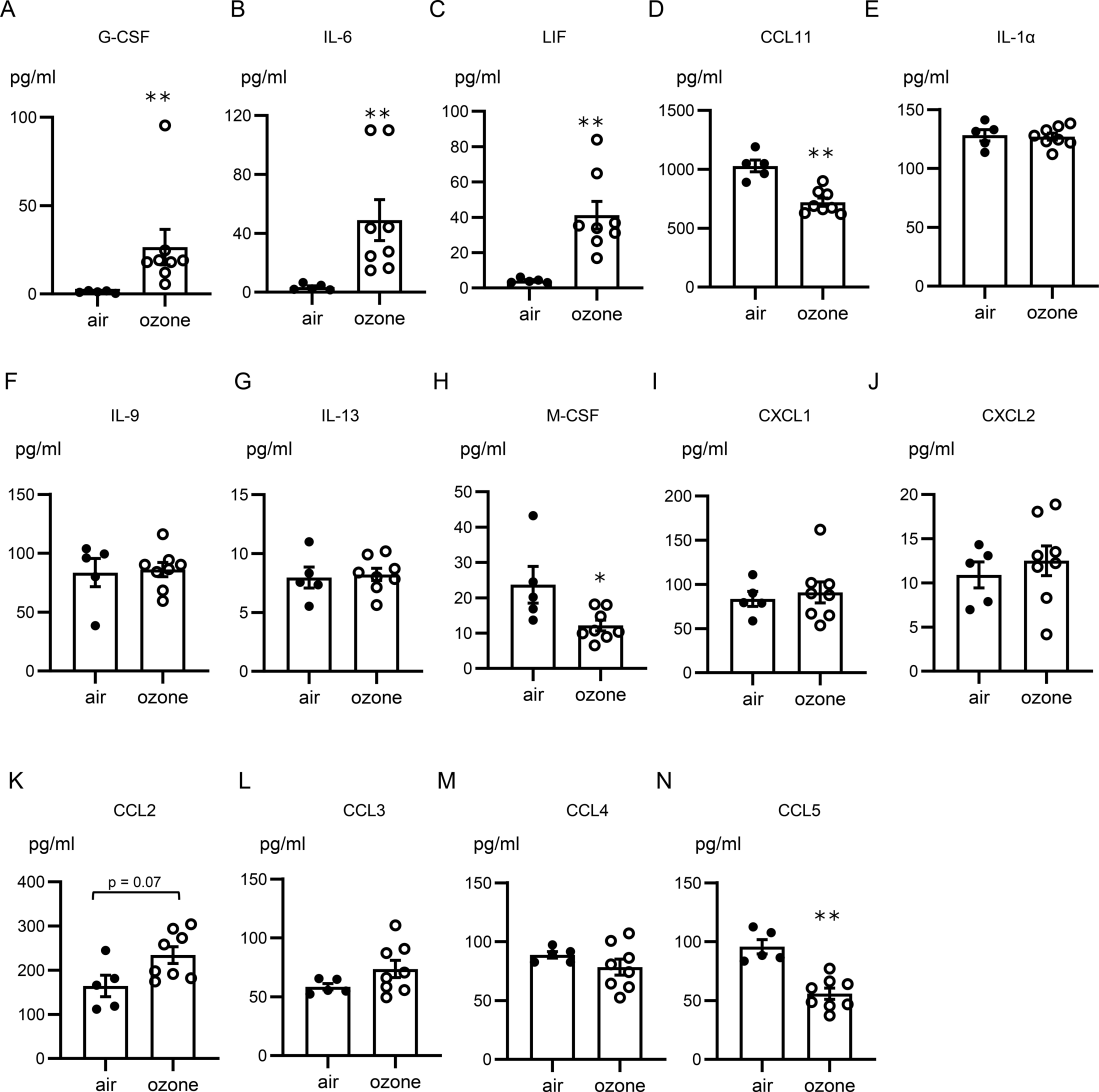
Concentrations of cytokines and chemokines in lungs of mice exposed to air or ozone: **(A)** G-CSF, **(B)** IL-6, **(C)** LIF, **(D)** CCL11, **(E)** IL-1α, **(F)** IL-9, **(G)** IL-13, **(H)** M-CSF, **(I)** CXCL1, **(J)** CXCL2, **(K)** CCL2, **(L)** CCL3, **(M)** CCL4, and **(N)** CCL5. The data shown are pooled from multiple experiments (n = 5–7 per group). Results are means ± standard error. **p < 0.01, *p < 0.05 compared with mice exposed to air. G-CSF, granulocyte colony-stimulating factor; IL-6, interleukin 6; LIF, leukemia inhibitory factor; CCL, C-C motif chemokine ligand; M-CSF, macrophage colony-stimulating factor; CXCL, C-X-C motif chemokine ligand.

To assess the alterations of cytokine and chemokine levels in lungs with ozone exposure, the results of multiplex assay data in the lungs of mice exposed to air or ozone were compared. The concentrations of granulocyte colony stimulating factor (G-CSF), IL-6, and leukemia inhibitory factor (LIF) were significantly higher in lungs of mice exposed to ozone than in those of mice exposed to air. C-C motif chemokine ligand 2 (CCL2) tended to be higher in mice exposed to ozone than in mice exposed to air, but the difference was not significant. CCL11, macrophage colony stimulating factor, and CCL5 were significantly lower in mice exposed to ozone than in mice exposed to air. Concentrations of other cytokines and chemokines including IL-1α, IL-9, IL-13, and C-X-C motif chemokine ligand 1 (CXCL1), CXCL2, CCL3, and CCL4 were not altered in the lungs of mice exposed to air and of mice exposed to ozone (**Figures 2A–N**).

### 3.3 Single-cell RNA sequencing analysis demonstrated that gene expression of osteopontin derived from dendritic cells was increased by ozone exposure

**Figure 3:**
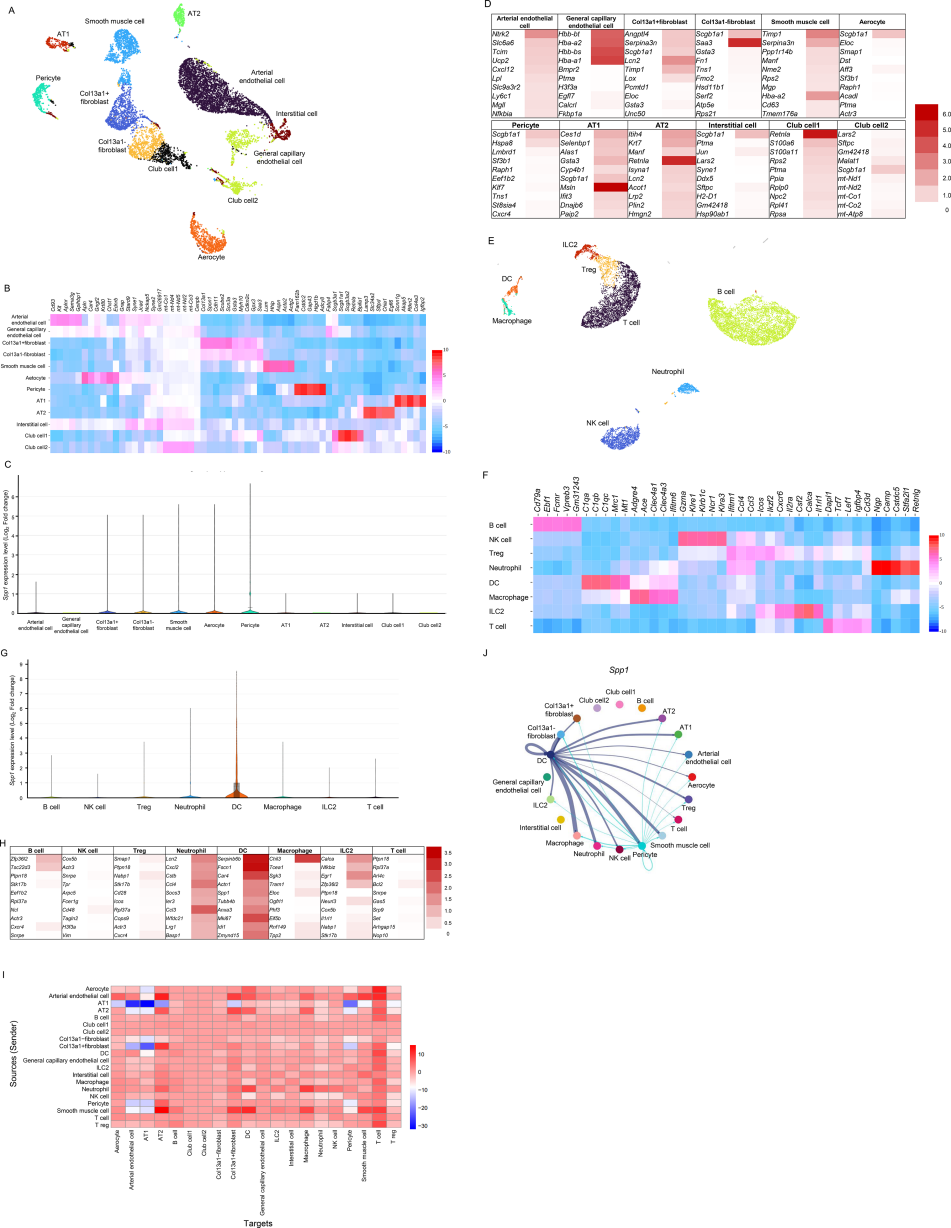
Gene expression and cell–cell communication analysis of CD45-negative and CD45-positive cells on single-cell RNA sequencing analysis in mice exposed to air or ozone. In CD45-negative cells, **(A)** annotated cell clusters with the algorithm of the Uniform Manifold Approximation and Projection method, **(B)** top 5 upregulated gene expressions depending on the p-value of targeted cell clusters compared with the entire dataset on a heat map indicating the log_2_ fold change values representing the differential gene expression levels, **(C)** expression levels of *spp1* in each cell cluster on a violin plot indicating the log_2_ fold change values representing the differential gene expression levels of *Spp1* between the selected cluster and all other clusters combined, and **(D)** top 10 genes significantly expressed in mice exposed to ozone compared with mice exposed to air on a heatmap indicating the log_2_ fold change values representing the differential gene expression levels. In CD45-positive cells, **(E)** annotated cell clusters with the algorithm of the Uniform Manifold Approximation and Projection method, **(F)** top 5 upregulated gene expressions depending on the p-value of targeted cell clusters compared with the entire dataset on a heat map showing the log_2_ fold change values representing the differential gene expression levels, **(G)** expression levels of *spp1* in each cell cluster on a violin plot showing the log_2_ fold change values representing the differential gene expression levels of *Spp1* between the selected cluster and all other clusters combined, and **(H)** top 10 genes significantly expressed in mice exposed to ozone compared with mice exposed to air on a heatmap showing the log_2_ fold change values representing the differential gene expression levels. On the cell–cell communication analysis called CellChat, **(I)** signal strengths from each cell in vertical lines as sources to each cell in horizontal lines as targets shown on a heat map. The color scale indicates relative signal strength changes, with red representing increased signal strength and blue representing decreased signal strength. **(J)** The network diagram of *spp1* shows the predicted cell–cell communication between different cell types identified by single-cell RNA sequencing. Each node represents a specific cell type, and the lines represent predicted ligand–receptor interactions. The thickness of the lines indicates the strength of interactions, and colors distinguish the different cell types.

To explore which cell clusters produce osteopontin in the lung, single-cell RNA sequencing analysis of CD45-negative cells and CD45-positive cells was performed. In terms of the CD45-negative cells, the analysis identified 12 clusters including arterial endothelial cells, general capillary endothelial cells, col13a1-positive fibroblasts, col13a1-negative fibroblasts, smooth muscle cells, aerocytes, pericytes, alveolar type 1 (AT1) cells, alveolar type 2 (AT2) cells, interstitial cells, and 2 types of club cells (**Figure 3A**). The expression patterns of the following major marker genes, *Ptprc*, *Pecam1*, *Acta2*, *Pdgfra*, *Col13a1*, *Sftpc*, *Pdpn*, *Scgb1a1*, and *Pdgfrb* are shown in **Supplementary Figures 2A–I**. The representative top 5 gene markers of each cell cluster compared with the entire dataset are shown by heatmap in **Figure 3B**. *Spp1*, which is a gene of osteopontin protein, was not highly expressed in clusters of CD45-negative cells, even though pericytes expressed it slightly (**Figure 3C**). For the top 10 genes upregulated by ozone exposure in each cell cluster, *Ntrk2* in arterial endothelial cells, *Hbb-bt, Hba-a2, Hbb-bs*, and *Hba-a1* in general capillary endothelial cells, *Lcn2* in col13a1-positive fibroblasts, *Saa3*in col13a1-negative fibroblasts, *Timp1* and *Serpina3n* in smooth muscle cells, *Scgb1a1* in aerocytes, *Msln* in AT1 cells, *Retnla* in AT2 cells, and *Retnla* in club cells 1 were significant, but genes upregulated by ozone exposure in CD45-negative cells were relatively low (**Figure 3D**).

In terms of CD45-positive cells, the analysis identified 8 clusters, including B cells, natural killer (NK) cells, regulatory T cells, DCs, macrophages, ILC2s, and T cells (**Figure 3E**). The expression patterns of the following major marker genes, *Ptprc*, *Cd3e*, *Cd19*, *Ncr1*, *Itgax*, *Itgam*, *Foxp3*, and *Il1rl1* are shown in **Supplementary Figures 3A–H**. The representative top 5 gene markers of each cell cluster compared with the entire dataset are shown by heatmap in **Figure 3F**. *Spp1* was expressed in DCs, but no other cell clusters of CD45-positive cells (**Figure 3G**). In addition, *Cd44*, a gene of the important receptor of osteopontin, was expressed in neutrophils, macrophages, and DCs (**Supplementary Figure 2I**). Among the top 10 genes upregulated by ozone exposure in each cell cluster, those identified in the DC cluster (including *Spp1*) and the neutrophil cluster showed greater significance than those in other cell clusters, although *Zfp36l2* and *Tsc22d3* in B cells, *Chil3* in macrophages, and *Calca*, *Nfkbiz*, *Egr1*, and *Zfp36l2* in ILC2s were also upregulated by ozone exposure (**Figure 3H**).

In the cell–cell communication analysis (CellChat pipeline) for the signal strength of each cell cluster to whole cell clusters in CD45-negative cells and CD45-positive cells, almost all signals were positively enhanced by ozone exposure except for: AT1s to aerocytes, arterial endothelial cells, AT2s, Col13a1-positive fibroblasts, and pericytes; Col13a-positive fibroblasts to arterial endothelial cells, AT1s, and pericytes; pericytes to arterial endothelial cells and AT1s; and smooth muscle cells to AT1s (**Figure 3I**). In terms of the specific gene signal, the *Spp1* signal was derived from DCs and transmitted to macrophages along with col13a1-negative fibroblasts, neutrophils, pericytes, smooth muscle cells, regulatory T cells, AT1s, AT2s, and DCs. Notably, the signal was also derived from pericytes and transmitted to other cell clusters, although its strength was weak (**Figure 3J**).

### 3.4 Sub-clustering of dendritic cells shows that gene expression of osteopontin is derived from monocyte-derived dendritic cells

**Figure 4:**
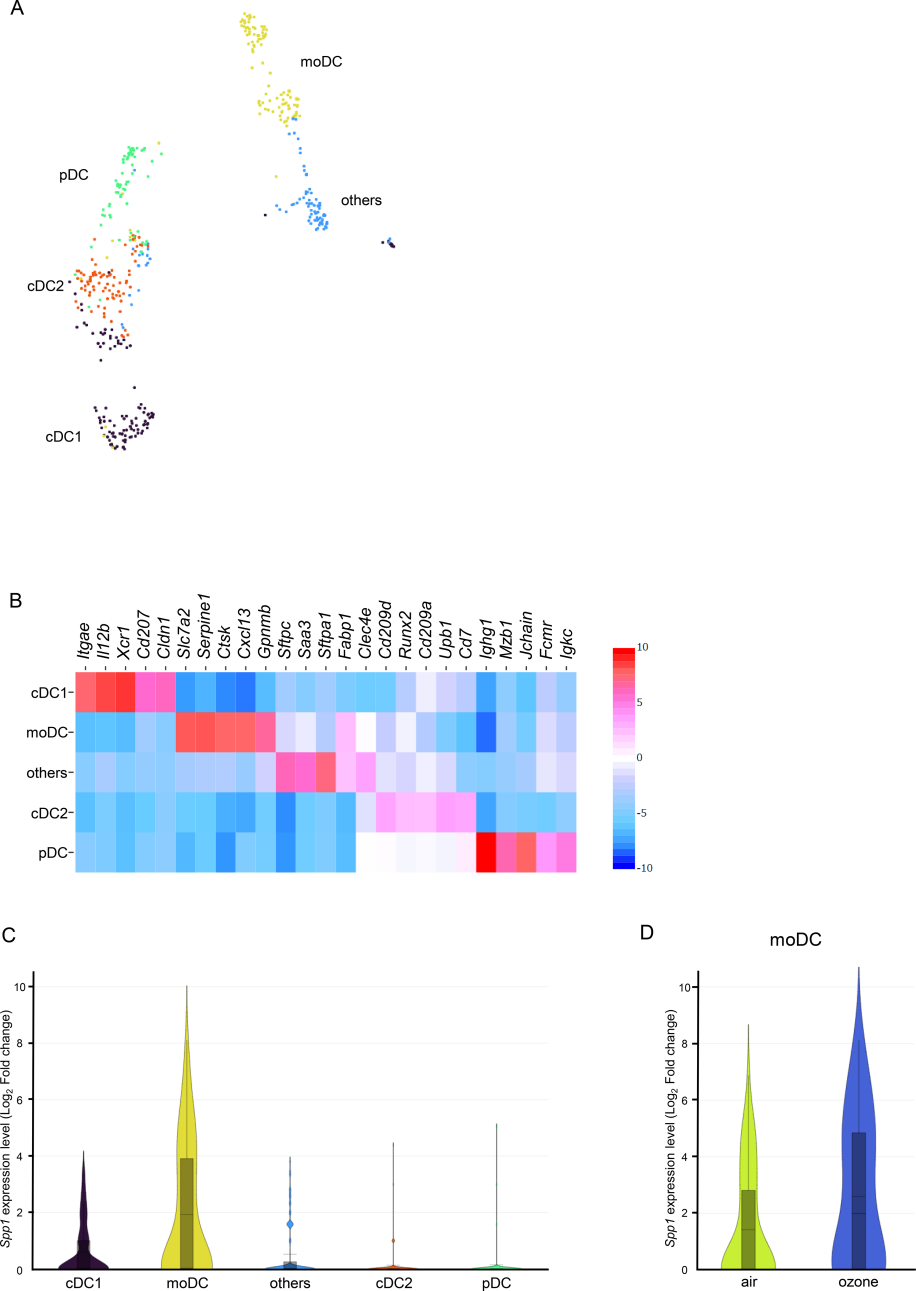
Sub-cluster analysis of dendritic cells on single-cell RNA sequencing analysis in mice exposed to air or ozone. **(A)** Annotated cell cluster with the algorithm of Uniform Manifold Approximation and Projection method, **(B)** top 5 upregulated gene expressions depending on the p-value of targeted cell clusters compared with the entire dataset on a heat map showing the log_2_ fold change values representing the differential gene expression levels, and **(C)** expression levels of *spp1* in each cell cluster on a violin plot showing the log_2_ fold change values representing the differential gene expression levels of *Spp1* between the selected cluster and all other clusters combined. **(D)** In moDCs, expression levels of *Spp1* in mice exposed to air or ozone are shown. cDC1, conventional dendritic cell type 1; moDC, monocyte-derived dendritic cell; cDC2, conventional dendritic cell type 2; pDC, plasmacytoid DC.

To explore which subcluster of DCs induces osteopontin, subcluster analysis of DCs was performed. The analysis identified 5 clusters including conventional DC type 1s (cDC1s), monocyte-derived DCs (moDCs), conventional DC type 2s (cDC2s), plasmacytoid DCs (pDCs), and others (**Figure 4A**). The expression patterns of the following major marker genes, *Itgax*, *Itgam*, *Batf3*, *Irf2*, *H2-Ab1*, *Tcf4*, and *Klf4* are shown in **Supplementary Figures 4A–G**. The representative top 5 gene markers of each dendritic subcluster compared with the entire dataset are shown by heatmap in **Figure 4B**. *Spp1* expression was higher in moDCs than in other subclusters of DCs (**Figure 4C**), and it was also higher in mice exposed to ozone than in mice exposed to air (**Figure 4D**), consistent with the previous results.

### 3.5 Ozone-induced neutrophilic airway inflammation and AHR are attenuated by administration of clodronate liposome

**Figure 5:**
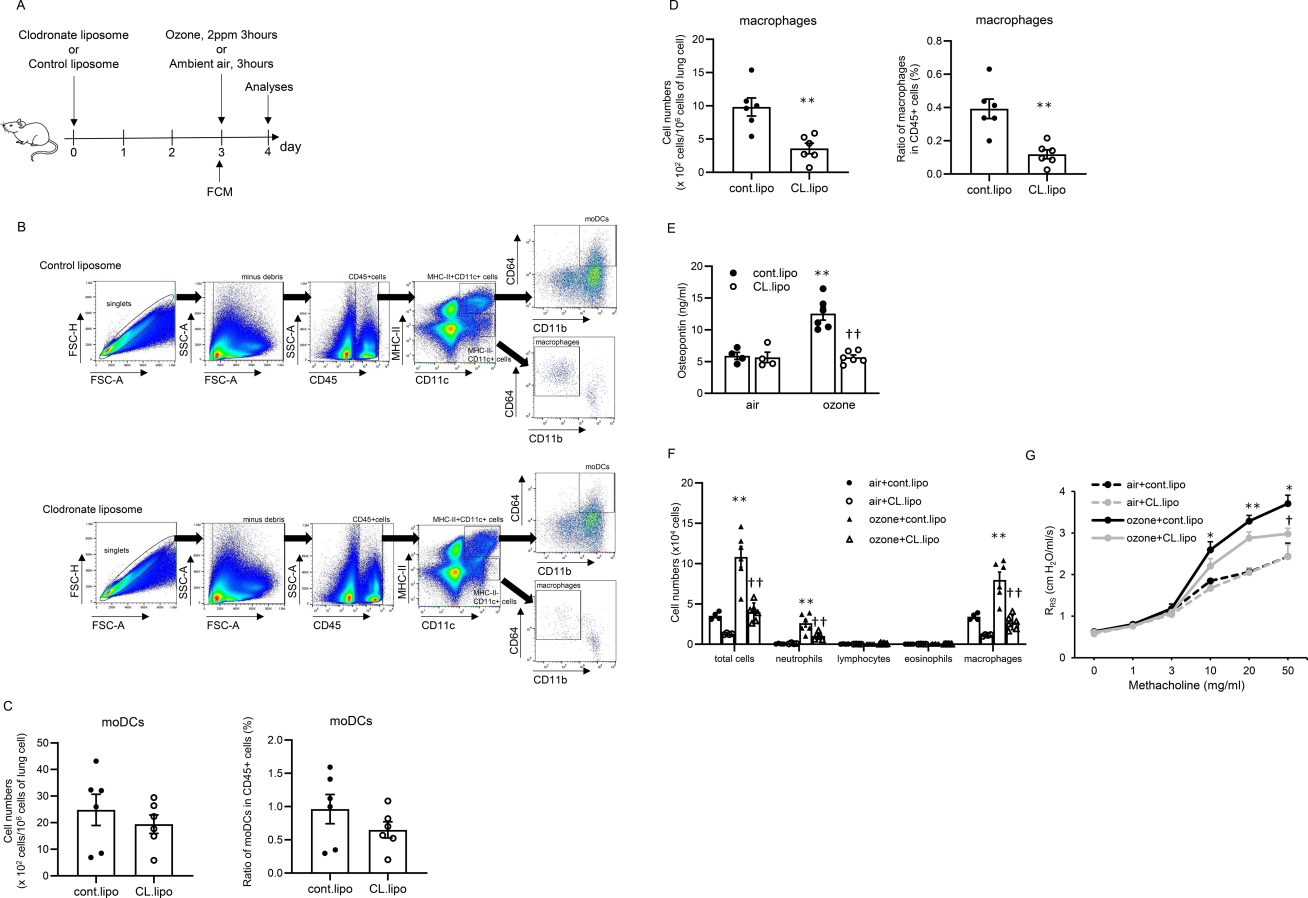
Cell counts of monocyte-derived dendritic cells and macrophages in lungs, osteopontin concentrations in lungs, cell counts in bronchoalveolar lavage fluid (BALF), and airway hyperresponsiveness in mice treated with control liposome or clodronate liposome and exposed to air or ozone. **(A)** Experimental protocol. **(B)** Flowcytometric analysis of single lung cells in mice treated with control liposome or clodronate liposome. **(C, D)** Ratios of moDCs and macrophages in lungs of mice treated with control liposome or clodronate liposome. **(E)** Concentrations of osteopontin in lungs of mice treated with control liposome or clodronate liposome and exposed to air or ozone. **(F)** Cell counts in BALF and **(G)** airway hyperresponsiveness in mice treated with control liposome or clodronate liposome and exposed to air or ozone. The data shown are pooled from multiple experiments (n = 4–6 per group). Results are means ± standard error. *p < 0.05, **p < 0.01 compared with mice exposed to air. †p < 0.05, ††p < 0.01 compared with mice exposed to ozone and treated with control liposome. FCM, flow cytometry; FSC, forward scatter; SSC, side scatter; moDC, monocyte-derived dendritic cell; cont.lipo, control liposome; CL.lipo, clodronate liposome.

To attenuate the function and cell numbers of moDCs, which are important cell clusters of *Spp1* signal-producing cells, as mentioned above (**Figures 3J**, **4C**), clodronate liposome was administered, referring to previous reports (30, 34–36) (**Figure 5A**). On flow cytometric analysis, cell numbers and ratios of moDCs defined by cell surface markers of CD45+MHCII+CD11c+CD11b+CD64+ (**Figure 5B**) in CD45 positive cells tended to be decreased, but the difference between mice treated with clodronate liposome and mice treated with control liposome was not significant (**Figure 5C**). Cell numbers and ratios of macrophages defined by cell surface markers of CD45+ MHCII-CD11c+CD64+CD11b- (**Figure 5B**) in CD45 positive cells were significantly lower in mice treated with clodronate liposome than in mice treated with control liposome (**Figure 5D**). In terms of the osteopontin concentration in the lungs, it was higher in mice treated with control liposome and exposed to ozone than in mice treated with control liposome and exposed to air. In mice exposed to ozone, the osteopontin concentration in the lungs was significantly lower in mice treated with clodronate liposome than in mice treated with control liposome (**Figure 5E**). For cell counts of BALF in mice treated with the control liposome, total cells, neutrophils, and macrophages were higher in mice exposed to ozone than in mice exposed to air. In mice exposed to ozone, these cells were significantly lower in mice treated with clodronate liposome than in mice treated with control liposome (**Figure 5F**). Histopathological analysis of the lungs showed no evidence of inflammatory cell infiltration or epithelial damage in the airways of either air-exposed or ozone-exposed mice treated with control liposome or clodronate liposome (**Supplementary Figure 5A**). In mice treated with control liposome, AHR was higher in mice exposed to ozone than in mice exposed to air, and in mice exposed to ozone, AHR was significantly lower in mice treated with clodronate liposome than in mice treated with control liposome (**Figure 5G**).

### 3.6 Ozone-induced AHR is attenuated by administration of an osteopontin inhibitor in mice

**Figure 6:**
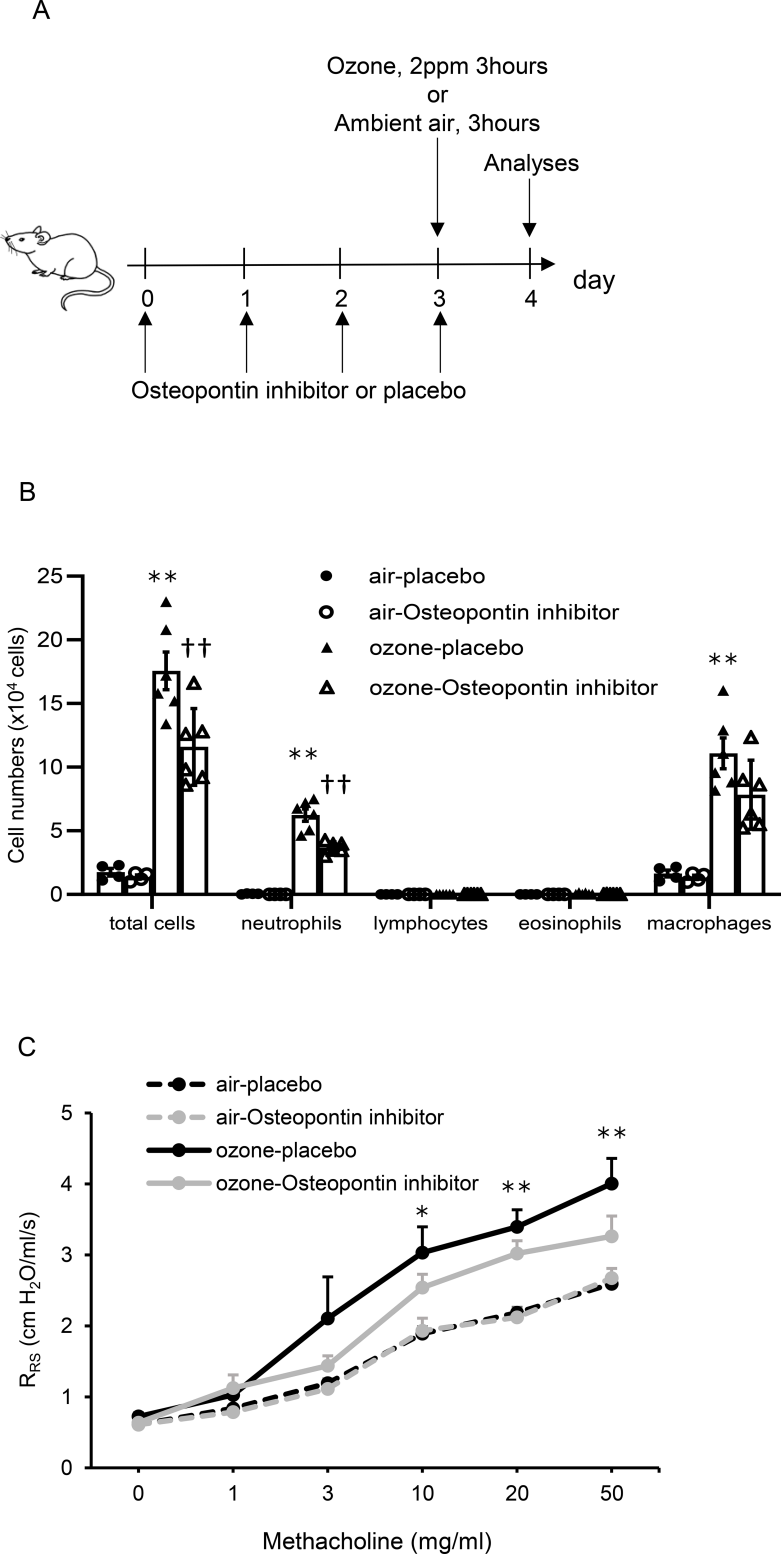
Cell counts in bronchoalveolar lavage fluid (BALF) and airway hyperresponsiveness in mice treated with placebo or osteopontin inhibitor and exposed to air or ozone. **(A)** Experimental protocol. **(B)** Cell counts in BALF of mice treated with placebo or osteopontin inhibitor and exposed to air or ozone. **(C)** Airway hyperresponsiveness in mice treated with placebo or osteopontin inhibitor and exposed to air or ozone. The data shown are pooled from multiple experiments (n = 4–6 per group). Results are means ± standard error. *p < 0.05, **p < 0.01 compared with mice exposed to air. ††p < 0.01 compared with mice exposed to ozone and treated with placebo.

To clarify the impact of an osteopontin inhibitor on ozone-induced pulmonary responses, mice exposed to air or ozone were treated with placebo (mouse IgG2c isotype control) or an osteopontin inhibitor (anti-mouse osteopontin antibody) (**Figure 6A**). In mice treated with placebo, cell numbers, including total cells, neutrophils, and macrophages in BALF, were significantly higher in mice exposed to ozone than in mice exposed to air. In mice exposed to ozone, total cells and neutrophils in BALF were significantly lower in mice treated with an osteopontin inhibitor than in mice treated with placebo (**Figure 6B**). Histopathological analysis of the lungs showed no evidence of inflammatory cell infiltration or epithelial damage in the airways of either air-exposed or ozone-exposed mice treated with placebo or an osteopontin inhibitor (**Supplementary Figure 6A**). In mice treated with placebo, AHR was significantly higher in mice exposed to ozone than in mice exposed to air. In mice exposed to ozone, AHR tended to be lower in mice treated with an osteopontin inhibitor than in mice treated with placebo, but the difference was not significant (**Figure 6C**).

### 3.7 Osteopontin induces upregulation of *NDUFA1* gene expression in bone marrow neutrophils

**Figure 7:**
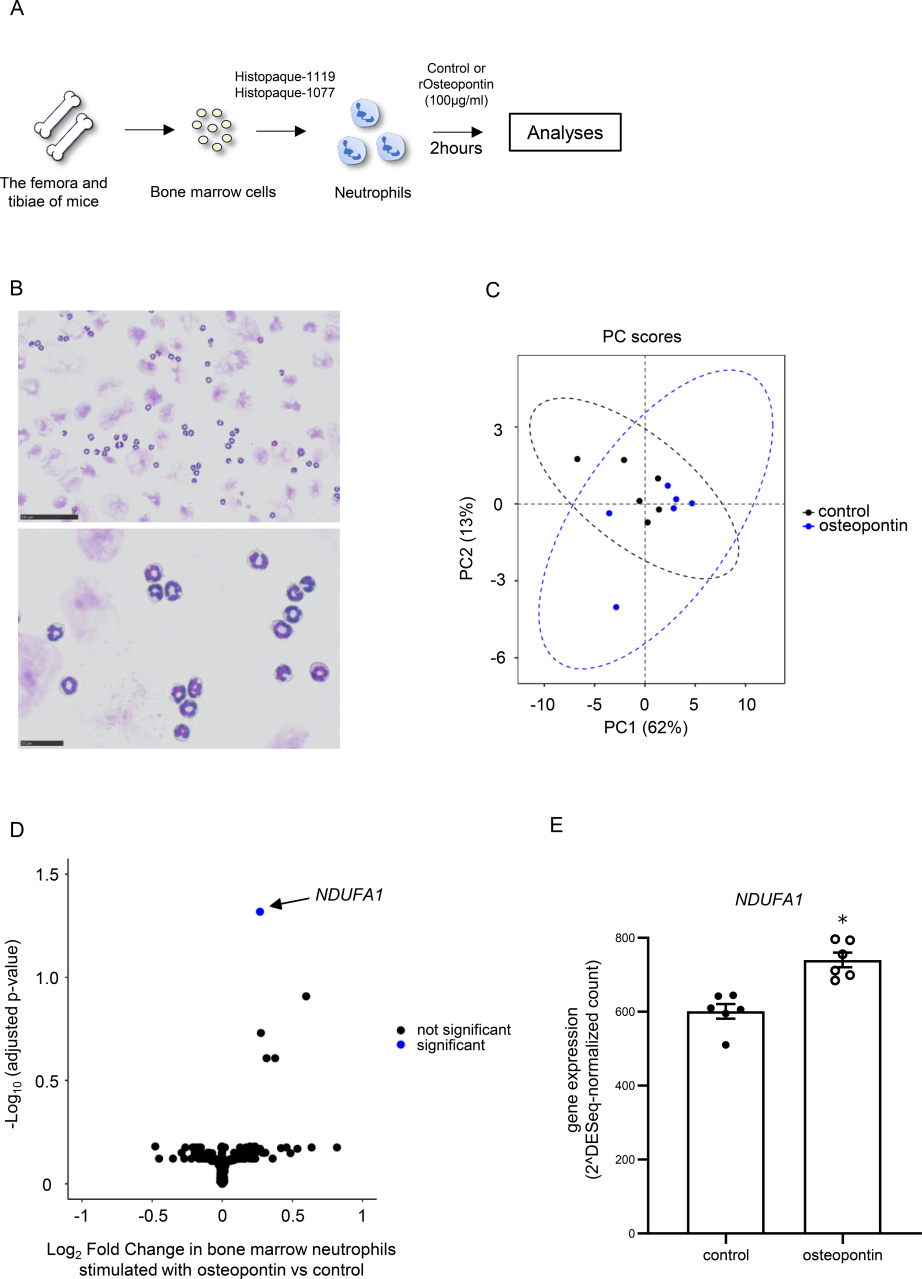
RNA sequencing and protein analyses of bone marrow neutrophils stimulated by osteopontin. **(A)** Experimental protocol of purification of bone marrow neutrophils. **(B)** Morphological analysis of bone marrow neutrophils by Diff-Quik staining. **(C)** Principal component scores with the confidence ellipsoids in bone marrow neutrophils with or without stimulation by osteopontin. **(D)** Volcano plots for differential expression analysis in bone marrow neutrophils with or without stimulation by osteopontin. **(E)** Gene expression of *NDUFA1* in bone marrow neutrophils with or without stimulation by osteopontin. The data shown are pooled from 6 samples per group. Results are means ± standard error. *p < 0.05 compared with the result of bone marrow neutrophils without stimulation.

To clarify the mechanistic impact of osteopontin for neutrophils, a comprehensive gene analysis by RNA sequencing and protein analysis of adhesion molecule expression by flow cytometry were performed with or without osteopontin stimulation *in vitro* (**Figure 7A**). First, the cells were visually confirmed to be neutrophils by microscopy with Diff-Quik staining and shown to consistently contain more than 90% neutrophils in viable cells (**Figure 7B**). For the principal features of gene expression in bone marrow neutrophils with or without stimulation by osteopontin, the confidence ellipsoids of the principal component scores showed overlapping distributions between neutrophils with osteopontin stimulation and those without stimulation, even though the areas were not completely identical (**Figure 7C**). Exploratory evaluation of pathway enrichment analysis showed that signals related to oxidative phosphorylation, aerobic respiration, cellular respiration, the nucleoside phosphate biosynthetic process, cytoplasmic translation, the purine ribonucleoside triphosphate metabolic process, the ribonucleoside triphosphate metabolic process, and proton motive force-driven mitochondrial adenosine triphosphate (ATP) synthesis were activated by osteopontin stimulation in neutrophils (**Supplementary Figure 7A**). Volcano plots for differential expression analysis showed that several genes were upregulated by osteopontin, and only one gene, *NDUFA1*, was significantly upregulated in neutrophils by osteopontin stimulation (**Figures 7D, E**). Expressions of adhesion molecules including CD11b, CD11a, and CD49d on neutrophils were not altered by osteopontin stimulation (**Supplementary Figures 7B–D**).

## 4 Discussion

The present results demonstrated that neutrophilic airway inflammation, AHR, and cytokines and chemokines in lungs, which were augmented by ozone exposure, were induced along with elevation of osteopontin concentrations in lungs. *Spp1*, a gene of osteopontin protein, was derived from DCs, especially moDCs, and its expression was upregulated by ozone exposure, with a confirmed signal to macrophages along with col13a1-negative fibroblasts, neutrophils, pericytes, and smooth muscle cells on single-cell RNA sequencing analysis. In addition, treatment with clodronate liposome, which can affect the cell activity of moDCs in terms of osteopontin production, attenuated neutrophilic airway inflammation and AHR augmented by ozone exposure, with decreased osteopontin concentrations in the lungs. Finally, specific abrogation of osteopontin by treatment with an osteopontin inhibitor attenuated ozone-induced neutrophilic airway inflammation, but not AHR, which highlighted the possibility that osteopontin has direct functional impact for neutrophils. The exploratory mechanistic analysis of neutrophils stimulated by osteopontin showed that upregulation of *NDUFA1* gene, but not adhesion molecules, in neutrophils might contribute to ozone-induced neutrophilic airway inflammation via osteopontin.

The present data showed the effect of osteopontin on augmentation of airway inflammation and AHR, which are pivotal in the pathophysiology of asthma, with elevation of cytokines including G-CSF, IL-6, and LIF in lung tissue. Notably, these cytokines were also increased in BALF by ozone exposure, as previously reported (16). Interestingly, neutrophilic inflammation and AHR were more pronounced at 24 hours after ozone exposure, but not at 48 hours. In addition, a previous report showed that AHR was not altered 4 hours after ozone exposure, even though neutrophil counts in BALF were increased compared with the result at 24 hours after ozone exposure (16). These data suggest that the 24-hour time point is appropriate for evaluating ozone-induced neutrophilic airway inflammation and AHR, which is consistent with previous reports describing time-dependent changes in respiratory barrier injury and inflammation (37). However, the present histological findings of the lungs did not show morphological changes due to ozone exposure. The discrepancy between increased neutrophils in BALF and the absence of obvious infiltration on H&E staining likely reflects compartment differences. In this acute ozone model, neutrophils primarily localize to the airway and alveolar luminal spaces, rather than accumulating in peribronchial or interstitial tissues. Because BALF examination detects luminal cells more sensitively than routine histology, mild and diffuse neutrophilia may not be evident on standard H&E sections. In contrast to the present model of acute stimulation using a single ozone exposure, multiple or long-term ozone exposure in mice, representing a chronic stimulation model, leads to distinct inflammatory phenotypes such as COPD-like features, which may have different effects on osteopontin induction in the lungs (38). In a meta-analysis of 706 patients with asthma and 332 healthy controls, osteopontin protein expression was higher in patients with asthma than in controls (39). Others also reported that, in serum and BALF samples, osteopontin protein expressions were significantly higher in patients with asthma than in controls. In this study, bronchial samples were obtained by endobronchial biopsy, and osteopontin-positive cells, which were expressed as epithelial and subepithelial cells of patients with asthma, were correlated with asthma severity and reticular basement membrane thickness, reflecting airway remodeling (24). In mice, ovalbumin (OVA)-induced AHR, goblet cell hyperplasia, and collagen deposition in the basement membrane were attenuated in osteopontin-deficient mice compared with wild-type mice (40). In addition, blockade of osteopontin in the sensitized phase attenuated and in the challenged phase augmented OVA-induced AHR and inflammation via regulation of T helper type 2-suppressing plasmacytoid DCs (41). Similar to the present results, Barreno et al. reported that the osteopontin level in BALF was increased with positive cells in alveolar macrophages by ozone exposure, and osteopontin-deficient mice showed significant attenuations of ozone-induced AHR and neutrophilic airway inflammation (42). These data suggested that osteopontin might contribute to asthma pathophysiology including AHR and airway inflammation with type 2 high and non-type 2 airway inflammation, consistent with the present results. Furthermore, a previous study suggested the potential of targeted therapies, including osteopontin and related molecules. For example, the humanized anti-osteopontin monoclonal antibody ASK8007 has been evaluated in patients with rheumatoid arthritis and was reported to be safe and well tolerated overall; however, it did not demonstrate robust clinical efficacy compared with placebo (43). In addition to these targets, ozone-induced lung injury is driven by oxidative stress–dependent epithelial cell death, including oxeiptosis, which leads to epithelial barrier disruption and the release of damage-associated molecular patterns (DAMPs) and alarmins such as IL-1α and IL-33. These danger signals subsequently activate innate immune pathways, including toll-like receptors, DNA-sensing mechanisms, inflammasomes, and the IL-1–MyD88 axis, thereby amplifying sterile inflammation and promoting chronic airway remodeling (44).

Importantly, the present single-cell RNA sequencing data showed that osteopontin, which was upregulated by ozone exposure, was derived from DCs, especially moDCs, and the signal was via macrophages along with col13a1-negative fibroblasts, neutrophils, pericytes, and smooth muscle cells (**Figures 3G, H, J**, **4C, D**). Osteopontin is thought to be produced by various immune cells, and it activates other cell clusters to modulate inflammation. For example, osteopontin is secreted by follicular CD153^+^ senescence-associated T cells and is functionally a negative regulator of apoptotic cell clearance (45). Others also reported that osteopontin is expressed in DCs to amplify production of IL-17A from CD4-positive cells, which is consistent with the present results as a source of osteopontin (46). Osteopontin also affects subtypes of DCs to produce Th1-associated inflammatory cytokines and chemokines and contributes to macrophage migration and activation in an *in vivo* model and cell line experiments (47–49). In addition, moDCs have a crucial role in the pathophysiology of asthma, which is demonstrated by increasing cell numbers of moDCs on stimulation by house dust mite and induction of Th2 responses by inhalation of diesel exhaust particles via CCR2-induced moDC recruitment (50, 51). According to these data, augmentation of ozone-induced airway inflammation and AHR by elevation of osteopontin derived from moDCs in the present data is consistent. Notably, ozone-induced pulmonary responses were attenuated in mice treated with clodronate liposome compared with mice treated with placebo, even though the absolute numbers and ratios of moDCs in lung were not significantly decreased (**Figures 5C, F, G**). In this model, the function of moDCs to produce osteopontin might be weakened without direct depletion of the cells by treatment with clodronate liposome as the mechanism. This idea was supported by the finding that *Spp1* was only expressed in moDCs in the present single-cell RNA sequencing analysis, and the osteopontin level in lungs was decreased by treatment with clodronate liposome, even though clodronate can also affect the cell activity of macrophages and other dendritic cell subtypes (34–36).

In the present study, osteopontin affected neutrophilic airway inflammation and might have had a direct interaction with neutrophils on cell–cell communication analysis (**Figure 3J**). Therefore, osteopontin stimulation may result in a mechanistic interaction to induce activation and migration of neutrophils. Koh et al. demonstrated that osteopontin contributed to recruitment and migration of neutrophils in mice; however, expression of adhesion molecules such as CD11b, CD11a, and CD49d on bone marrow neutrophils was not altered by direct stimulation with osteopontin in the present study (**Supplementary Figures 7B–D**). Recent clinical data of patients with sepsis showed that the ratio of neutrophil extracellular traps (NETs) was associated with poor outcomes (30-day and 90-day deaths) and the serum osteopontin level. In that study, NETs in the neutrophils purified from healthy donors were induced by direct stimulation with osteopontin *in vitro* (52). According to these data, osteopontin might be involved in neutrophil activation, which is supported by the dominant gene expression of *Cd44*, a crucial receptor of osteopontin on neutrophils along with DCs and macrophages (**Supplementary Figure 3I**) (53, 54). In addition, exploratory evaluation of pathway enrichment analysis showed that signals related to oxidative phosphorylation, aerobic respiration, cellular respiration, and proton motive force-driven mitochondrial ATP synthesis activate neutrophils by osteopontin stimulation (**Supplementary Figure 7A**). In the life cycle of neutrophil metabolization, mature neutrophils are considered to activate mitochondrial metabolism in response to specific stimuli, highlighting the critical role of oxidative phosphorylation in supporting effector functions such as formation and migration of NETs (55). These activation pathways may converge on mitochondrial bioenergetic processes, including oxidative phosphorylation and proton motive force-driven ATP synthesis, thereby sustaining aerobic respiration and the energy supply required for ROS production and degranulation. Thus, osteopontin likely serves as a link between extracellular signaling and mitochondrial metabolic reprogramming in neutrophils. Importantly, a gene, *NDUFA1*, was significantly upregulated by stimulation with osteopontin of bone marrow neutrophils on RNA sequencing analysis in the present study (**Figures 7D,F**). This gene codes MWFE protein and is essential for functional complex 1 in mammalian mitochondria and thus related to oxidative phosphorylation for ATP production (56). Hence, *NDUFA1* might be associated with the activity and function of neutrophils stimulated by osteopontin, which possibly induces neutrophilic airway inflammation in mice.

The present study has limitations. First, only male mice were used in the present study, and whether the present results can be generalized to female mice cannot be determined, because no data were collected from female mice in the present experimental protocol, and it was previously reported that pulmonary responses to ozone differed between male mice and female mice (13, 28). Second, the log_2_ fold change of *NDUFA1* in osteopontin-stimulated bone marrow neutrophils was low, and, thus, its biological relevance remains uncertain despite its statistical significance. Third, the source of osteopontin was assessed solely based on RNA sequencing data, and confirmation at the protein level was not performed. Fourth, other ozone exposure conditions, such as different concentrations or repeated exposures, were not examined, which may yield results different from those observed in the present study.

## 5 Conclusions

Ozone exposure increased osteopontin levels in the lungs, leading to enhanced neutrophilic airway inflammation and AHR. Single-cell RNA sequencing identified DCs, especially moDCs, as the main source of *Spp1*, with additional interactions with macrophages, neutrophils, fibroblasts, pericytes, and smooth muscle cells. Clodronate liposome treatment decreased osteopontin expression and attenuated ozone-induced neutrophilic inflammation and AHR. Moreover, specific inhibition of osteopontin suppressed neutrophilic airway inflammation, but not AHR, suggesting a direct functional role of osteopontin in neutrophil regulation.

## Data Availability

The datasets presented in this study can be found in online repositories. The names of the repository/repositories and accession number(s) can be found in the article/materials and methods.
