# Supplementary material for: Osteopontin from monocyte-derived dendritic cells mediates ozone-induced pulmonary responses in mice
Source: Front Immunol. 2026 Mar 19;17:1748667. doi: 10.3389/fimmu.2026.1748667 (PMC13044518; doi:10.3389/fimmu.2026.1748667)
Supplement: Supplementary file 1 [file DataSheet1.docx]

Supplementary Material


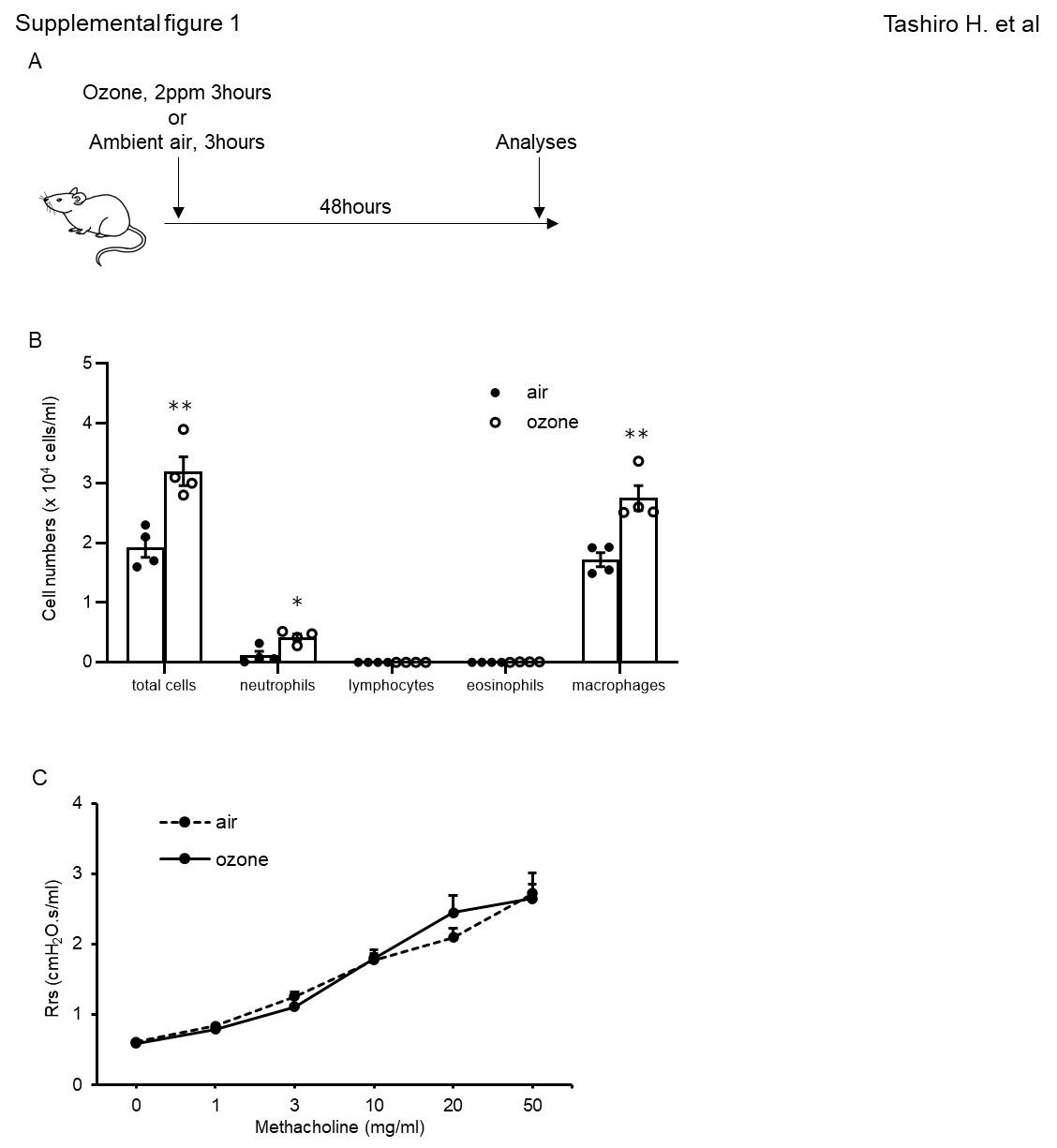


Figure S1

Cell counts in bronchoalveolar lavage fluid (BALF), airway hyperresponsiveness, and concentrations of osteopontin in lungs of mice exposed to air or ozone at 48 hours after the exposures. (A) Experimental protocol. (B) Results of cell counts in BALF in mice exposed to air or ozone. (C) Results of airway hyperresponsiveness in mice exposed to air or ozone. The data shown are pooled from multiple experiments (n = 4 per group). Results are means ± standard error. **p < 0.01, *p < 0.05 compared with mice exposed to air.


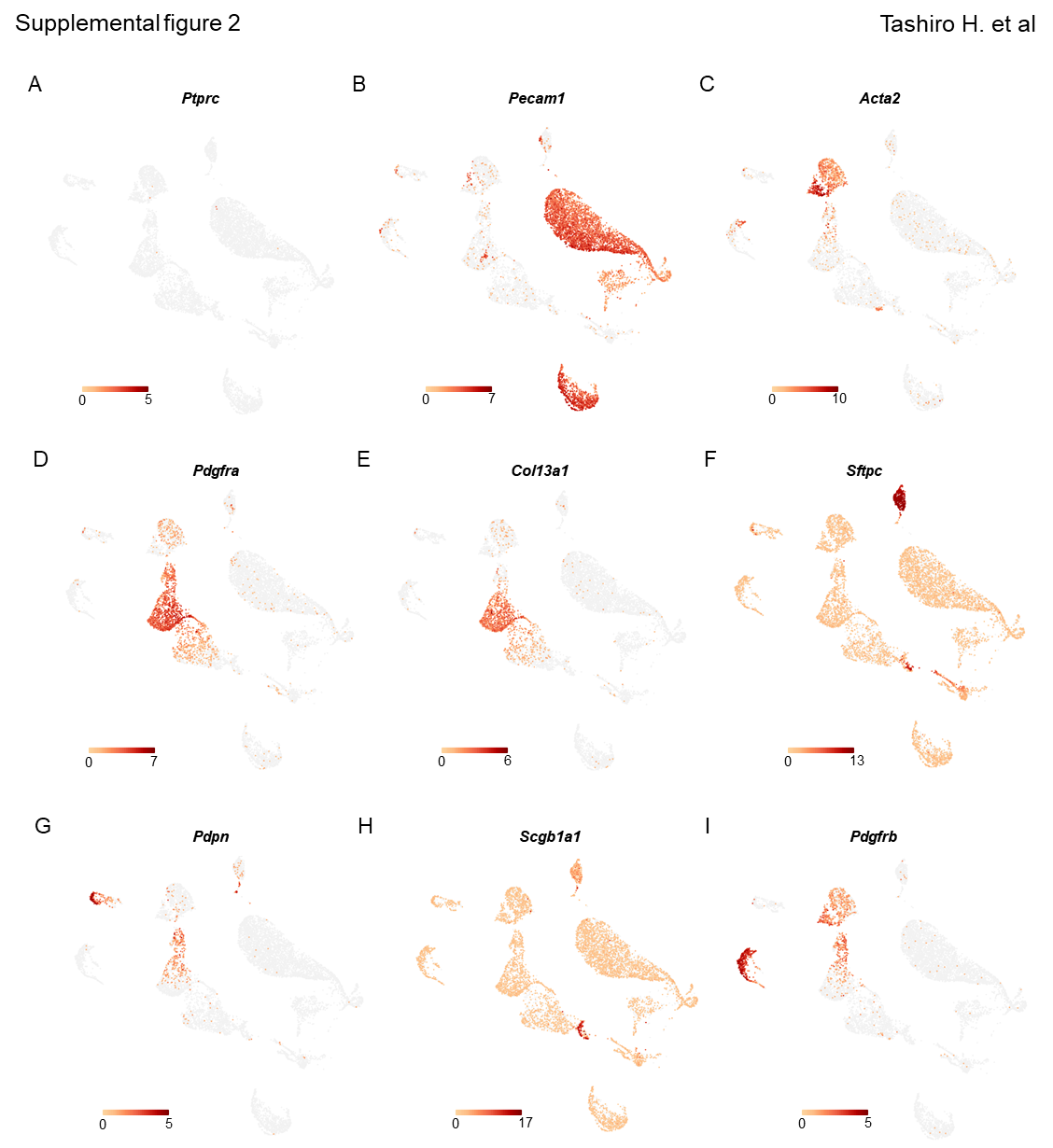
Figure S2

Gene expression analysis in CD45-negative cells of lung on single-cell RNA sequencing analysis in air or ozone exposed mice. Gene expressions of (A) *Ptprc*, (B) *Pecam1*, (C) *Acta2*, (D) *Pdgfra*, (E) *Col13a1*, (F) *Sftpc*, (G) *Pdpn*, (H) *Scgb1a1*, and (I) *Pdgfrb*.


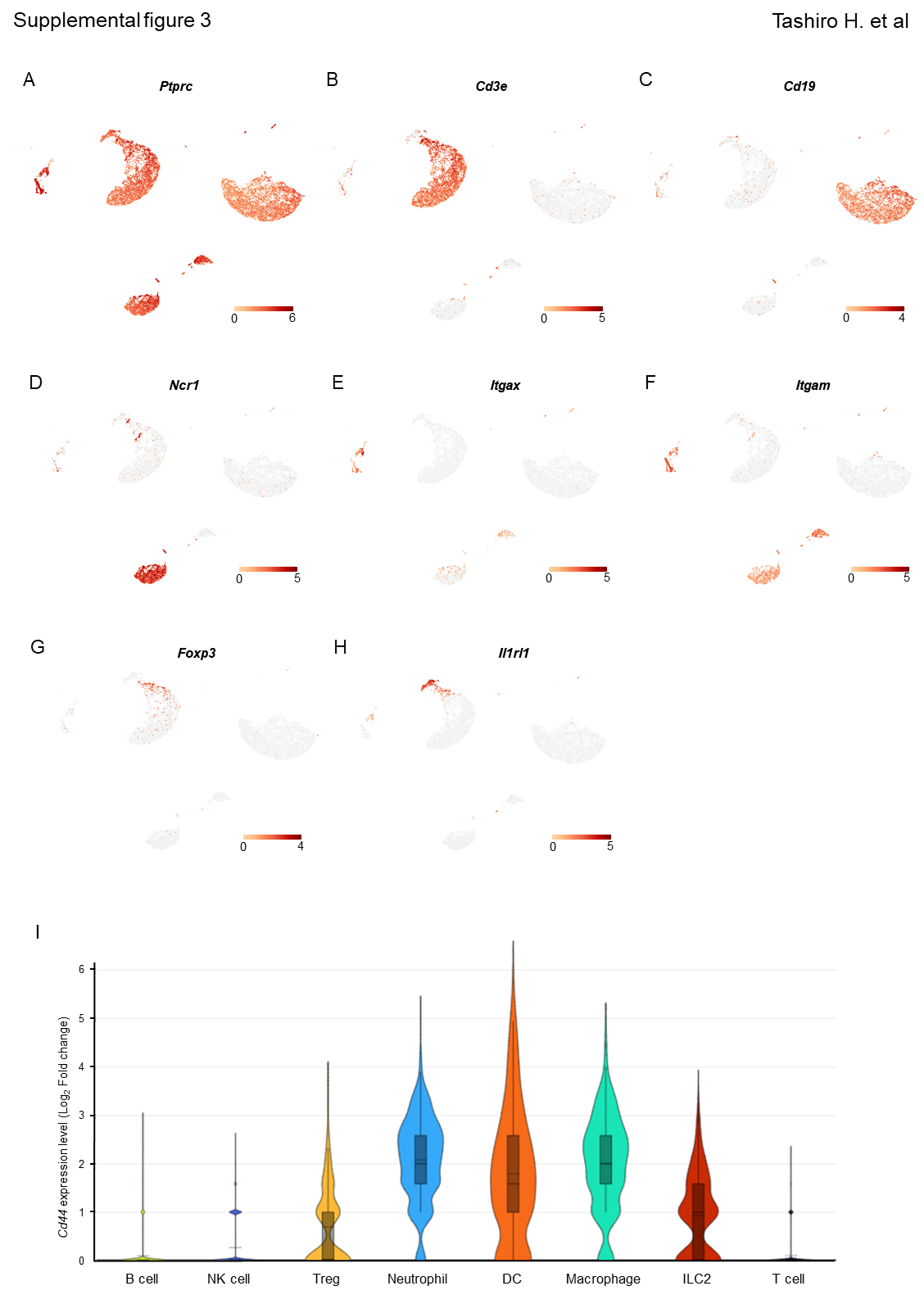
Figure S3

Gene expression analysis in CD45-positive cells of lung on single-cell RNA sequencing analysis in air or ozone exposed mice. Gene expressions of (A) *Ptprc*, (B) *Cd3e*, (C) *Cd19*, (D) *Ncr1*, (E) *Itgax,* (F) *Itgam*, (G) *Foxp3*, and (H) *Il1rl1*. (I) Expression of *Cd44* in each cell cluster on a violin plot. NK cell: natural killer cell, Treg: regulatory T cell, DC: dendritic cell, ILC2: group 2 innate lymphoid cell.


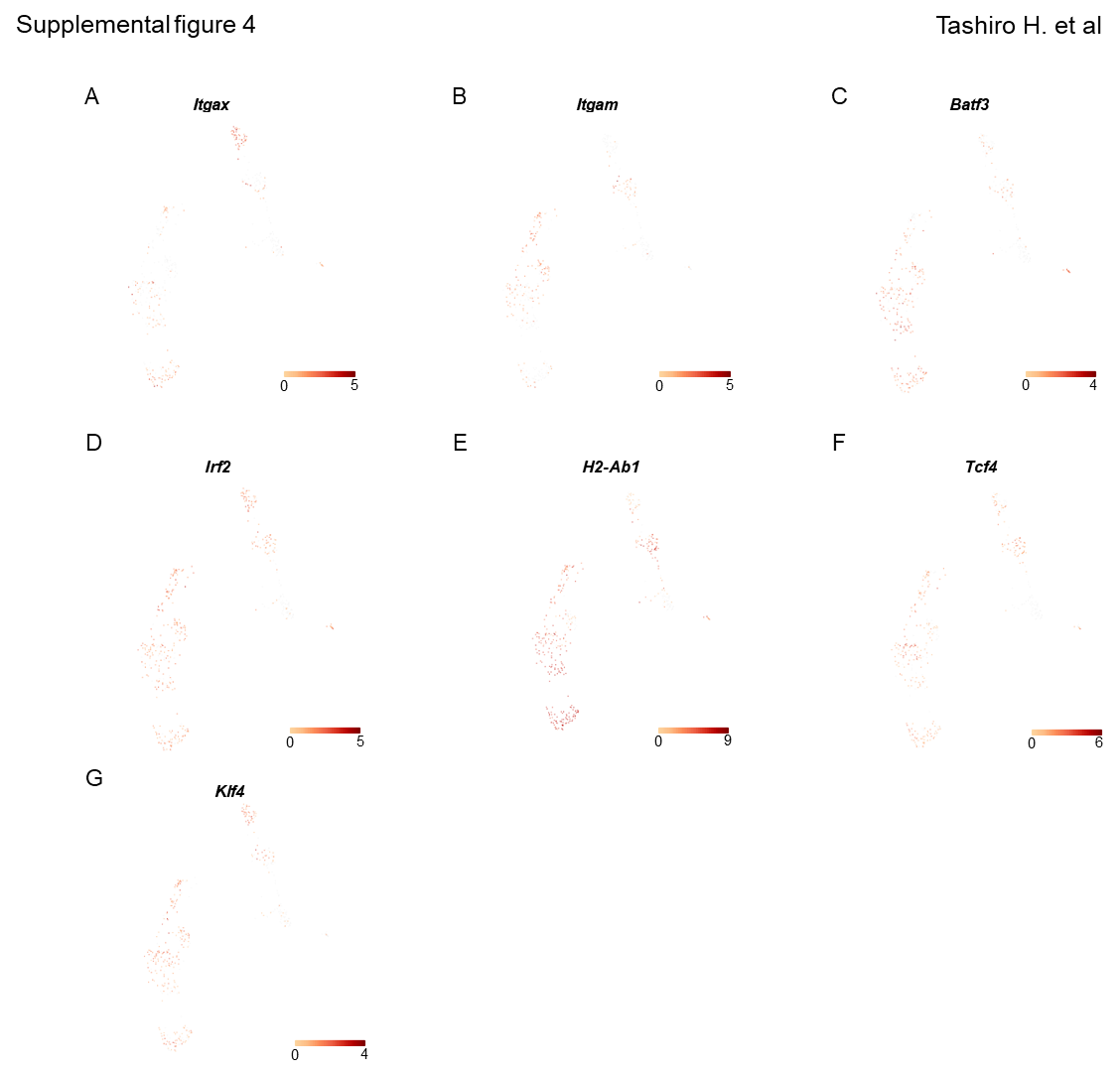


Figure S4

Gene expression analysis of distinct clusters in dendritic cells on single-cell RNA sequencing analysis in air or ozone exposed mice. Gene expressions of (A) *Itgax*, (B) *Itgam*, (C) *Batf3,* (D) *Irf2*, (E) *H2-Ab1*, (F) *Tcf4*, and (G) *Klf4*.


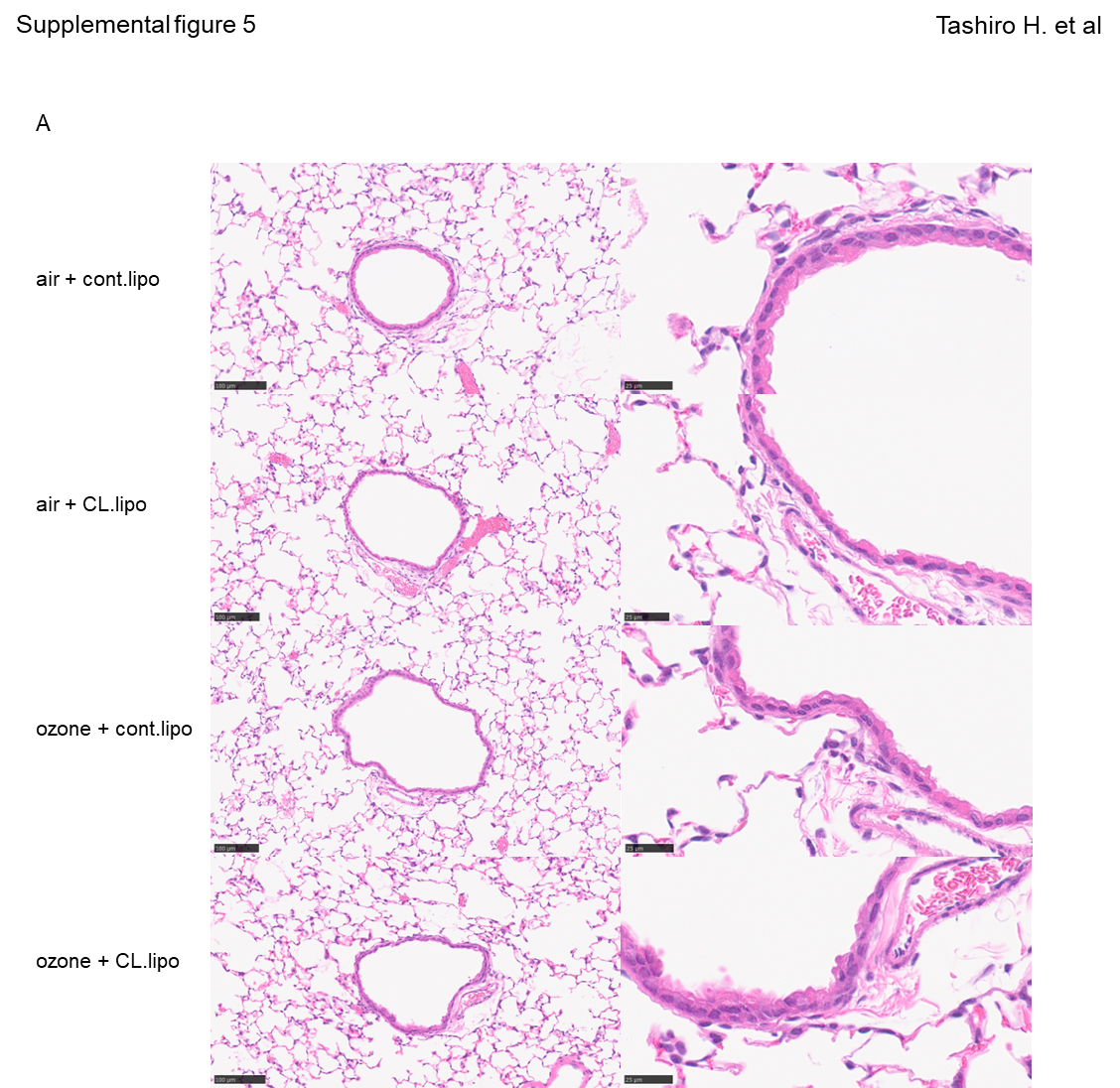


Figure S5

(A) Lung histological examination by hematoxylin and eosin staining in mice treated with control liposome or clodronate liposome and exposed to air or ozone. cont.lipo: control liposome, CL.lipo: clodronate liposome


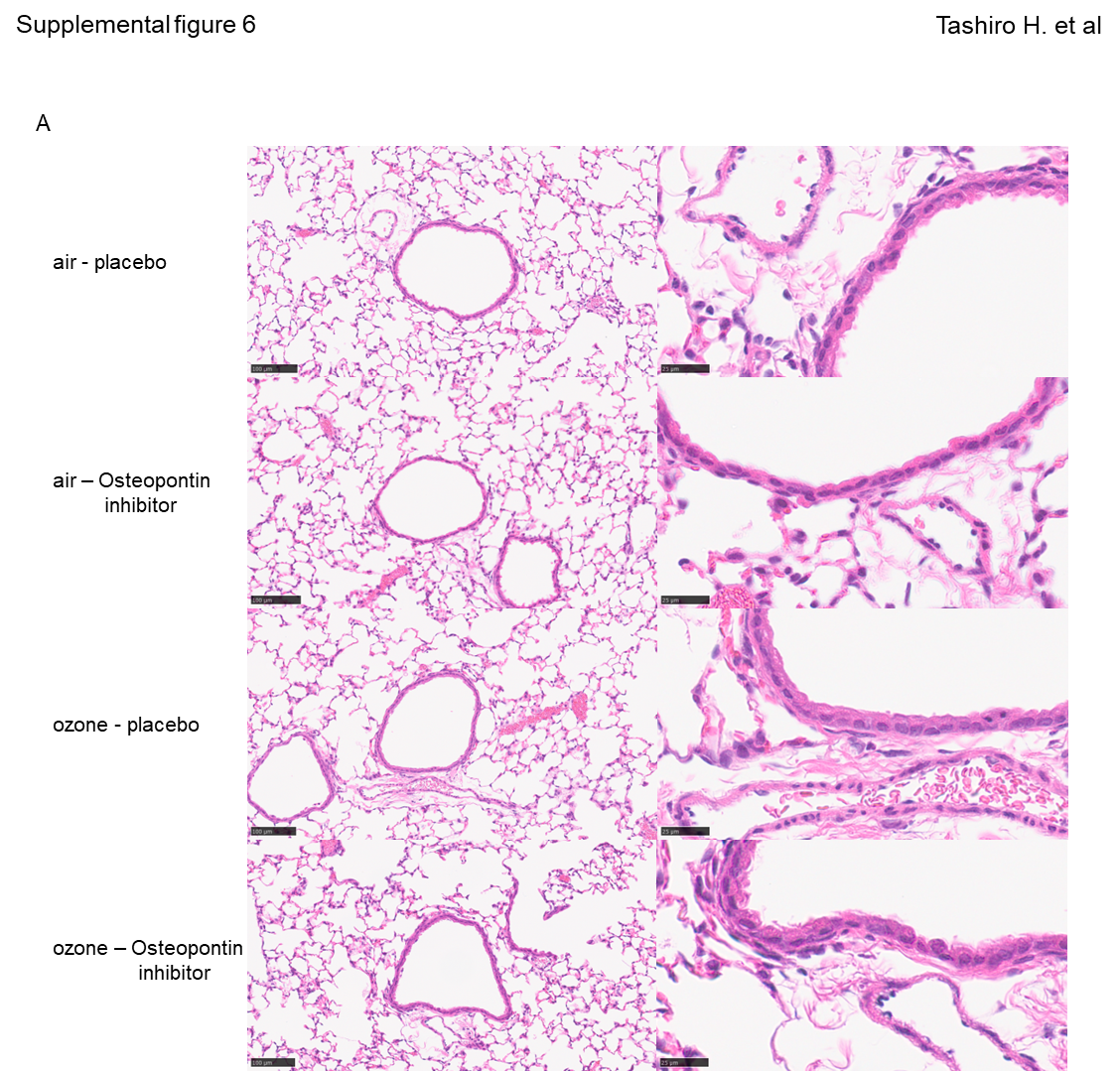


Figure S6

(A) Lung histological examination by hematoxylin and eosin staining in mice treated with placebo or osteopontin inhibitor and exposed to air or ozone.


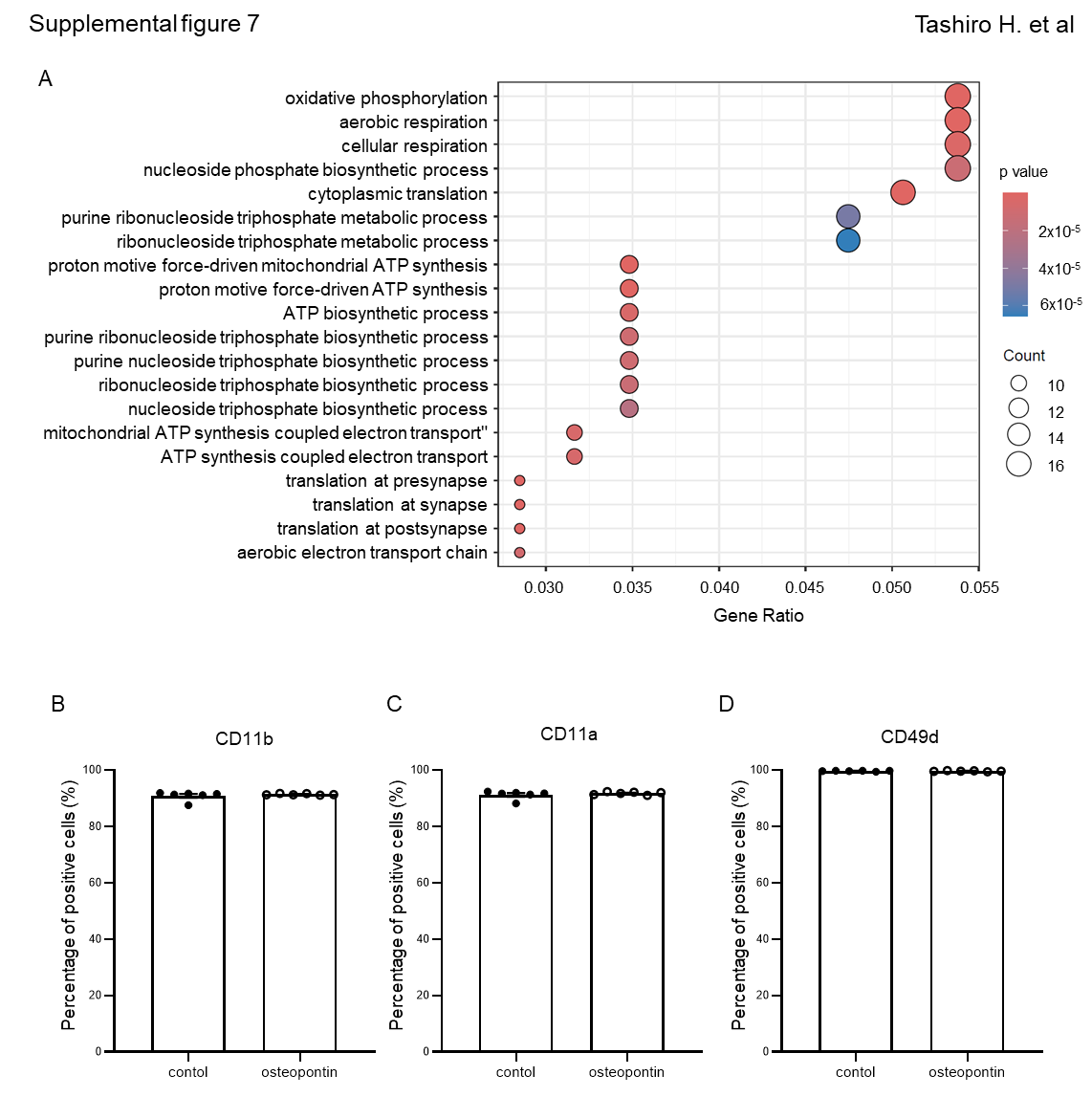


Figure S7

(A)Exploratory pathway enrichment analysis of bone marrow neutrophils stimulated by osteopontin on RNA sequencing analysis using genes with a nominal p < 0.05 considered significant. (B-D) Ratio of CD11b, CD11a and CD49d positive cells in bone marrow neutrophils with or without stimulation of osteopontin.
